# Insights and Experiences From the ‘Gatekeepers’: A Qualitative Study Exploring Clinician Perspectives on Providing Publicly Funded Prenatal Exome Sequencing

**DOI:** 10.1111/ajo.70028

**Published:** 2025-03-26

**Authors:** Samantha Dayman, Melissa Graetz, Lisa Hui, Lilian Downie

**Affiliations:** ^1^ Department of Obstetrics, Gynaecology, and Newborn Medicine The University of Melbourne Parkville Australia; ^2^ Genetics in the Northeast Mercy Hospital for Women Heidelberg Australia; ^3^ Reproductive Epidemiology Murdoch Children's Research Institute Parkville Australia; ^4^ Victorian Clinical Genetics Service Murdoch Children's Research Institute Parkville Australia

**Keywords:** clinician perspectives, exome sequencing, genomics, prenatal diagnosis, utility

## Abstract

**Background:**

Genomics has improved etiological diagnosis for foetal structural anomalies. It is being increasingly utilised in prenatal investigation both in Australia and internationally. To date, literature reporting diagnostic yield according to indication has been available. There is limited literature around the challenges of implementation and other aspects of utility.

**Aims:**

We aimed to explore the experiences and perspectives of clinicians involved with the delivery of a state‐wide public prenatal exome sequencing (pES) service in Australia.

**Materials and Methods:**

This qualitative study was developed using a pragmatism framework. A multidisciplinary cohort of clinicians across all tertiary foetal medicine units in Victoria was interviewed. Inductive content analysis was used to understand the experiences, impact, and utility of pES.

**Results:**

Eight clinician interviews were analysed. The impact of pES on clinicians included: increased pressure, higher emotional toll, and balancing the benefits with resource limitations. PES was most useful when it provided prognostic information. The clinicians felt that pES had the most utility for patients when the result informed their decision about whether or not to continue a pregnancy. Clinicians acknowledged their ‘gatekeeper’ role and valued a collaborative, multidisciplinary approach. The main perceived harm for patients was the anxiety associated with waiting times for results.

**Conclusions:**

This study provides insights into the delivery of a publicly funded pES program. Our findings highlight the importance of the multidisciplinary team in the successful implementation of genomic technologies in reproductive health.

## 1 Introduction

Genetic and genomic diagnostic technologies have revolutionised clinical practice in reproductive health, especially in the evaluation of foetal structural anomalies [1]. Foetal structural anomalies are detected by ultrasound in up to 5% of pregnancies [2], and first‐tier genetic tests (karyotyping, Fluorescent In Situ Hybridisation and chromosomal microarray analysis) reveal a genetic cause in up to 40% of cases [3]. Prenatal exome sequencing (pES) is indicated when a multidisciplinary team with genetic expertise believes the foetal phenotype has a genetic aetiology [4]. Large cohort studies [5, 6] investigating foetal structural anomalies in 234 and 610 pregnancies, respectively, found an increased diagnostic yield from pES of ~9%–10%. Subsequent systematic reviews and meta‐analyses reported pooled diagnostic yields for pES of 31%, varying from 2%–53% depending on foetal phenotype, study design and type of structural anomalies [3].

Increasingly, the importance of personal utility is recognised, particularly in the prenatal setting. This refers to the ‘ability of a test result to be used for decisions, actions or understanding relevant to the patient beyond healthcare‐related management, including facilitating future planning and facilitating reproductive planning’ [7]. Studies have found that pES was useful for determining prognoses for pregnancies impacted by structural anomalies and informing prenatal and neonatal management and family planning [8, 9, 10]. It helped families prepare for the arrival of a child with medical needs [11], and informed prenatal counselling around termination of pregnancy or neonatal palliative care [12]. There is also recognised value in uninformative pES results as they can support parents' decision to continue a pregnancy [9, 13, 14]. Personal utility needs to be balanced with diagnostic yield when determining when testing is performed.

While international studies have examined parent experiences of pES [15, 16, 17], few studies have explored the views and experiences of healthcare professionals directly involved in pES. The limited literature outlines positive experiences in relation to timely diagnosis and better counselling [18] but reports challenges, including managing anxiety while awaiting results, appropriate case selection, and building a skilled workforce [19]. These issues mirror those identified in the introduction of genomics into other high‐acuity settings such as critically ill neonates [20].

The state Department of Health in Victoria, Australia, has provided funding for pES since 2019 through tertiary foetal medicine units, with specific eligibility criteria: high likelihood of a monogenic condition, testing in a prenatal setting has utility, and the requirement for a multidisciplinary team (MDT) discussion. The aims of this study were to explore the experiences and perspectives of clinicians involved with the delivery of the Victorian Clinical Sequencing Initiative.

## 2 Materials and Methods

### 2.1 Study Design

This exploratory study adopted a qualitative methodology to ensure a range and depth of clinician experiences and perspectives were captured. This allowed for flexibility in understanding the nuances of responses, factoring in different professions and health services where pES is being delivered. The study was designed using a pragmatism framework, an approach that focuses on ‘practical understandings’ of concrete, real‐world issues [21]. The exploration of clinicians' experiences and perspectives was well served by this approach as pragmatism values various viewpoints and perspectives of stakeholders in developing a deeper understanding of the subject matter [22].

### 2.2 Setting

Publicly funded pES is available to patients at four metropolitan Melbourne tertiary hospitals (Health Services). To proceed with funding of pES, a minimum of 3 specialist clinicians must review the case against eligibility criteria. For a case to be eligible for public funding, there must be agreement that the diagnosis is likely to be identified using a genomic test and that the condition is severe, involving a complex neurological condition or affecting multiple systems. If a case does not reach consensus, it is not eligible for funding. If there is disagreement, then eligibility can be determined on a case‐by‐case basis, and the decision is recorded as one made with uncertainty.

There is an expectation that pES would inform current or future reproductive decision making for the patient/couple. Postpartum pES following stillbirth, including terminations of pregnancy, was also eligible if the results would inform future reproductive planning.

The Department of Health provides $AUD2500 for each eligible case. All prenatal samples are tested as trios when possible, and rapid turn‐around time provides results within 15 days. Testing is sequential, following chromosome microarray, in all but exceptional circumstances (such as very late gestation). Clinicians from the Health Services who were directly involved in managing at least five cases of pES were eligible to participate in the study. Purposive sampling was used to ensure sufficient sampling from each of the three relevant clinical specialties involved in patient management and care: genetic counsellors (GC), clinical geneticists (CG), and maternal–foetal medicine (MFM) specialists.

### 2.3 Recruitment

Coordinators of genetics at the four Health Services identified eligible participants and distributed the study invitation by email. After informed consent was provided, semi‐structured interviews were conducted via Zoom (Interview Topic Guide—see Appendix A). Interviews were audio‐ and video‐recorded and stored in the Zoom cloud along with the corresponding automated transcripts. The deidentified transcripts were uploaded to NVivo12 software (QSR International Pty Ltd., 2020) on a secure password‐protected Murdoch Children's Research Institute server. The profession of each participant was retained to assist with interpretation of findings.

### 2.4 Analysis

Interview transcripts were analysed using Inductive Content Analysis (ICA). ICA is an iterative method of summarising the content of a data set by identifying codes within the data that represent units of meaning. These codes emerge from the data, rather than being matched to a pre‐determined list of content items [23]. All transcripts were coded by SD and two transcripts were co‐coded by co‐authors (L.H. and L.D.) for rigour and consistency.

This study received ethics approval and governance authorisation from the Royal Children's Hospital Melbourne Human Research Ethics Committee (HREC Reference 90771/RHCM‐2023).

## 3 Results

Eight clinicians, of approximately 30 eligible, participated in the study, including three GC's two MFM, and three CG's. The interviews were conducted between April 2023 and August 2023; mean duration of interviews was 56 min.

**FIGURE 1:**
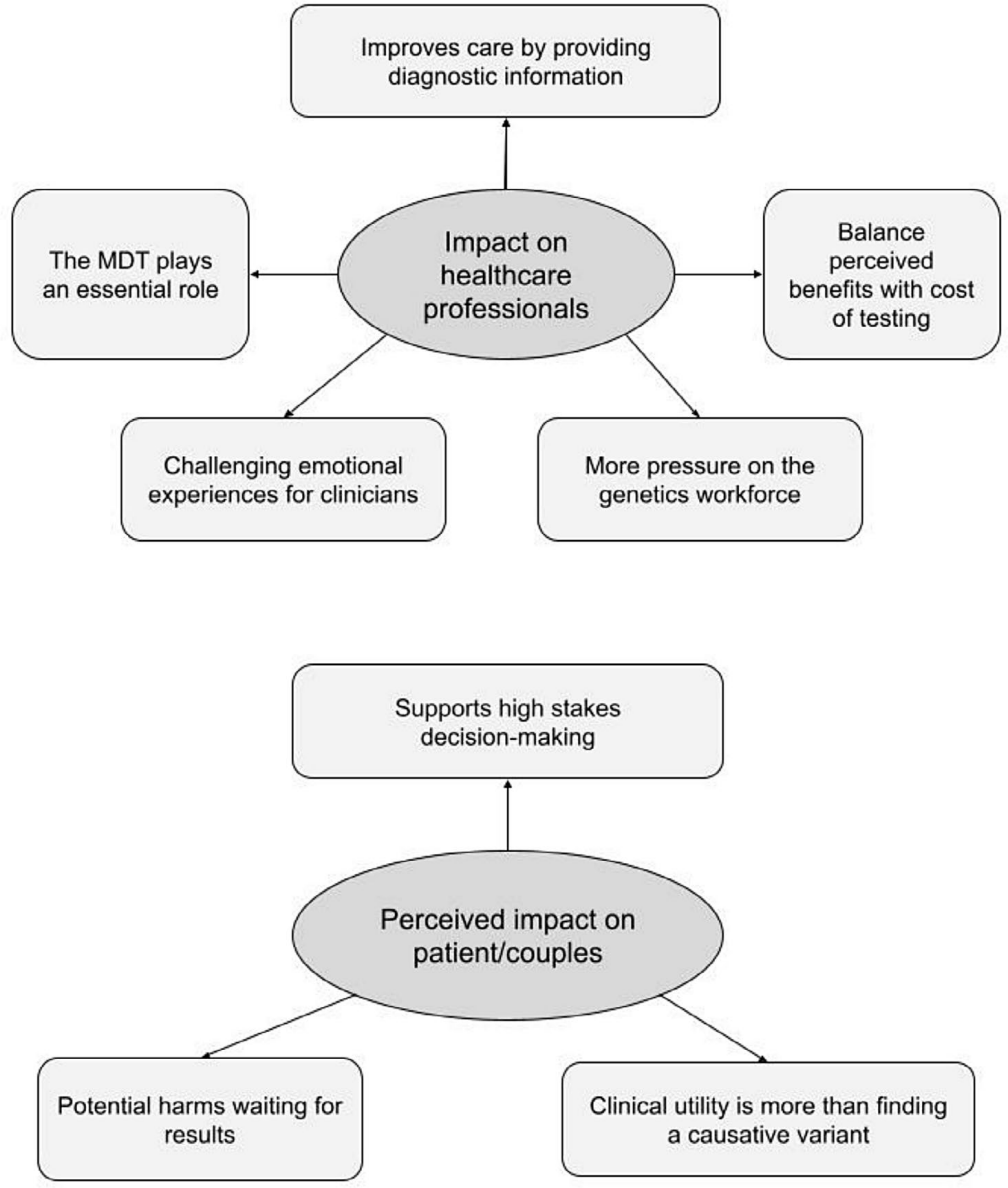
Main categories and subcategories of inductive content analysis.

Inductive content analysis revealed two main categories of findings: impact on healthcare professionals and perceived impacts on pregnant women/couples. Sub‐categories within these two themes have been identified and summarised in Figure 1.

### 3.1 Impact on Healthcare Professionals

#### 3.1.1 Improves Care by Providing Diagnostic Information

Clinicians highlighted that pES improves clinical care by providing more diagnostic information. Clinicians recognised value in providing clarity about the prognosis for the developing baby, recurrence risk, or management for the pregnancy, delivery and neonatal care.

#### 3.1.2 The MDT Plays an Essential Role

Clinicians identified the MDT as being key to the successful provision of pES. It facilitated appropriate decisions about patient selection, which was highly valued. The MDT also provided families with consistent information and continuity of care during their complicated pregnancy. Some clinicians recognised that continuity of care is difficult to deliver in the context of workload pressures. Others highlighted the benefits of collaborating with other health services to ensure the state‐wide delivery of pES was consistent, and to promote learning. ‘That [MDT]email dialogue is very helpful, because that's how we learn and kind of develop consensus and formulate ideas and they're very good practice for learning…’ (CG2). The role of nonmedical staff, namely genetic counsellors, midwives, and social workers was also highly valued for the wrap‐around support they provided to patients.

#### 3.1.3 Balance Perceived Benefits With Cost of Testing

Clinicians identified as having a gatekeeper role, and felt they must balance the perceived benefits to patients and clinical care with the cost of testing. ‘Part of the gatekeeper role we have is that we want to do good clinical practice, but a lot of it is financially driven and the fact that we can't afford to provide it to everyone. So, then it raises the question: Well, let's say it was free, or very, very cheap, how would that change what we would offer? And clearly, we would offer it a lot more liberally.’ (CG2).

#### 3.1.4 More Pressure on the Genetics Workforce

Clinicians expressed concerns about the impact of pES on the clinical workforce. These related to the increased administrative burden, and the urgency of pregnancy related testing. This time sensitivity led to personal experiences of stress by some clinicians. There was also worry about the downstream impacts these urgent cases had on waitlists for genetics referrals. ‘These are the conversations that we're having at the moment around resourcing and funding because we're doing more and more [pES], but we don't have any additional resources or funding for it. And they're the most urgent of the urgent cases. So, it just means that they get seen, and other people don't. And they're so complex that the actual appointment often takes a lot of time. Then prep for it, and the MDT discussion…and looking up all the relevant information. All of that takes a lot of time’. (GC2).

#### 3.1.5 Challenging Emotional Experiences for Clinicians

Clinicians revealed challenging emotions associated with delivering pES and their role in supporting families in high stakes decision‐making around continuing or terminating pregnancies where foetal structural anomalies had been identified. ‘It's both a terrifying and a powerful position to be in, where you realize that what you say is going to make an often, a life‐or‐death decision, if you will, or life or no life decision, for this family—is somewhat dictated upon what you say’. (CG1).

This emotional toll was a potential source of burnout for staff and seen as a potential threat to the long‐term sustainability of the clinical service. ‘That flow on effect [of the increased workload] is burnout for the workforce, and people feeling overloaded and stressed and anxious. And if we're not properly resourced for these things, then it just doesn't work’ (GC2).

### 3.2 Perceived Impact on Patient/Couples

#### 3.2.1 Supports High Stakes Decision‐Making

All clinicians acknowledged the benefits of pES for patients and couples who are making decisions about a pregnancy that is complicated by foetal structural anomalies. When pES confirms a genetic diagnosis, this can allow parents to make informed decisions, with greater certainty about what the expected outcomes could be for their unborn child. ‘[A benefit of pES is] being able to say to a couple ‘Here is a really devastating diagnosis, this is awful, here's what it means for your baby’, and them being able to say, ‘That's really helpful to inform my decision around what I do next’. So, I think for decision making purposes, when you have a diagnosis, it can be really instrumental for families’. (CG1).

Clinicians referred to the value of uninformative or negative findings from pES in providing reassurance to continue a pregnancy. Clinicians noted the importance of counselling families around negative results insofar as they do not guarantee the absence of a genetic basis for the clinical presentation. ‘And negative tests sometimes can be as valuable as a positive test [result] for families because it then confirms their belief that they want to keep going because there's nothing additional: “What I dealt with at the beginning, and what I was prepared to continue with in this pregnancy remains the same. You haven't added any additional findings and haven't worsened the prognosis”’. (MFM2).

#### 3.2.2 Potential Harms Waiting for Results

Clinicians raised concerns about the potentially harmful impact of pES on the pregnant woman and family by protracting the decision‐making process, especially when the results of pES do not provide additional information to inform decision‐making. This delay can create additional anxiety. Clinicians highlighted the importance of providing parents with ongoing support during this period of waiting. ‘So [the extra time for testing] does cost a lot of in terms of emotional issues to the family. Especially when the parents are not sure which way they want to go, it can take a bit of an emotional toll in many families. Some families do change their plan while waiting for the results. So once in a while we see families that say “Okay, it's too much for us. We don't want to continue the pregnancy”’. (CG3).

#### 3.2.3 Clinical Utility Is More Than Finding a Causative Variant

Clinicians had a broader view of the clinical utility of pES than simply identifying a causative genetic variant. Clinicians recognised that in many cases, a non‐diagnostic result can be just as valuable for parents, as this gives some patients more confidence to continue a pregnancy with a structural anomaly. ‘Traditionally, there's been a view in genetics that a high [diagnostic] yield means a higher clinical utility, but that doesn't necessarily follow in prenatal, particularly when there's really only one decision to be made and that is whether a couple will continue with a pregnancy or whether they will terminate a pregnancy’. (CG2).

Results were seen to contribute to decisions regarding obstetric management and neonatal care in the current pregnancy, as well as information to inform genetic counselling about reproductive risks and options for future pregnancies. Clinicians were optimistic that clinical utility of pES would improve with further experience and accumulation of knowledge. ‘As we have more experience about the longer‐term impacts (of not only a positive but also a negative exome) and how protective is it to have a negative exome, this will increase the precision of our counselling so that people can then make better informed decisions.’ (MFM2).

## 4 Discussion

This study provides insights into clinicians' perspectives and experiences associated with the delivery of publicly funded pES. Participants valued the contribution of pES to their counselling and clinical management, and reported that the main benefit was to inform a couple's decision to terminate or continue the pregnancy, reflecting the views of parents' experience of pES [15, 16, 18].

Importantly, clinicians emphasised that a nondiagnostic pES result could be valuable in providing reassurance to continue a pregnancy. The value provided by a nondiagnostic test is supported by literature describing parents experiences with pES, where these results can be perceived as ‘good news’ and families feel they have done all they could [10, 18, 24]. Our results also highlight the caution that should be taken in selecting appropriate families to offer testing and the need for patient centred counselling. In some situations, families may not tolerate the waiting for results and ultrasound findings may be adequate for families to make decisions [10].

One significant harm identified was the additional waiting period associated with pES, a concern described by others in the field [19]. In our setting, testing is performed after a nondiagnostic microarray result; however, with improvements in screening and the expanding abilities of genomic technology, turn‐around times may shorten [25]. Clinicians were concerned that extending the waiting period for pES results could add to parents' distress, especially when results are uninformative. Clinicians advocated for sufficient support mechanisms for families during this time and stressed the importance of the MDT, midwives, social workers, and genetic counsellors in providing this support.

While clinicians have consistently recognised the importance of patient counselling and support [18], this study uniquely highlighted the need for clinician support and the importance of having well‐established mechanisms such as professional supervision and clear guidelines for best practice to prevent burnout. The mismatch between the increased workload associated with the provision of pES and the allocation of funding for testing was an additional concern and is mirrored internationally [19, 26]. In addition, potential downstream impacts on patient care and meeting the needs of the growing number of patients referred to genetic services were highlighted. They advocated for more resources to build the genomics workforce to ensure the sustainability of pES and future advances in care, again a sentiment that has been described in previous studies [27].

While this study provides an insight into the challenges experienced by clinicians, it also describes features of the existing model of care that are positive. Clinicians highly valued the role of the MDT in patient selection and providing continuity and consistency of care. The MDT was also seen as a source of continuing education and peer support. Genetic counsellors were seen as central to the successful provision of pES, anchoring the myriad stakeholders involved (MDT members, laboratory staff, parents, and healthcare professionals in the wider community).

This study was only able to report the clinicians' perspectives on the impact of FES on the experiences of pregnant women/couples. Future research investigating the lived experience of patients in an Australian context would further inform the future delivery of pES and add to the emerging body of literature [19, 26, 27]. Participants in this study comprised a small, self‐selected sample in the Victorian context. A larger sample of healthcare professionals was not feasible given the small number of specialists working in this area. Limited previous literature describes a service where abortion laws have a large effect on service pressures and laboratory turn‐around time [9]. Understanding the experiences of other services and stakeholders (such as referring clinicians, medical scientists and policy makers) will assist in shaping future services to maximise the value from the resource limited health sector.

As the cost of sequencing decreases, it is likely to be more frequently incorporated into perinatal care [28]. It is thus imperative that there are sufficient and appropriately experienced staff available to deal with the expanding workload. Our study revealed some of the hidden costs of providing advanced genomic testing, including increased administrative and clinical workload and often a higher emotional toll and potential for staff burnout.

This study has provided valuable insights into the experiences and perspectives of clinicians involved in a clinical pES service. The clinicians see themselves as ‘gatekeepers’ of this expensive genomic technology, while supporting patients in a time‐sensitive, high‐stakes decision making environment. The greatest utility of pES (whether diagnostic or uninformative) was in informing patient decisions to continue or terminate a pregnancy. Collaboration and peer support within an MDT, and an adequately funded and resourced genetics workforce, were seen as essential for the successful and sustainable provision of pES within the public hospital system.

## Conflicts of Interest

The authors declare no conflicts of interest.

## Data Availability

Data available on request from the authors. The data that support the findings of this study are available from the corresponding author upon reasonable request.

## Appendix A

### Research Project: Interview Guide

*(Verbal Informed Consent to be confirmed prior to Interview:*

1. *Prior to Recording per Protocol*
2. *Confirmation with Participation after recording has commenced)*.

#### Start of Interview

Thank you for taking part in our research study. I’d like to start by confirming some details.

1. Could you please confirm:
  - Your profession
  - The hospital or service where you are located (when involved with the Fetal Exome Sequencing program)

It would be helpful to know more about your clinical role, especially as it relates to this program.

2 Could you please briefly describe your clinical role?
  - How many Fetal Exome Sequencing cases have you been involved with (approximately) and
  - When did you first become involved with this program of exome sequencing in the perinatal space?

While the focus of our research is your experience of Fetal Exome Sequencing in a clinical setting, it would also be helpful to know if you have any experience with exome sequencing in the perinatal field as part of research.

3 Have any of your experiences with FES been in the context of research?
  - Could you please describe these for me?

Our research is particularly interested in the experiences and perspectives of clinicians involved with Fetal Exome Sequencing in the clinical setting.

4 Could you please share your thoughts, in general, about the introduction of this technology into the clinical setting?
5 What are your impressions about the **benefits** of FES? We’d be interested to learn about the clinical benefits as well as the benefits to individual patients and their families, and other stakeholders that you think play an important role.

Check:

○ What are some advantages of FES when compared with existing technologies?
○ Could you provide any examples of where you have seen these benefits?

With the introduction of any new technology, **challenges** can be encountered.

6 I’d be interested to hear your impressions about any limitations or challenges associated with FES? Again, it would be helpful to understand this from your direct perspective, as well as any ways that these problems are experienced by patients and other stakeholders.

Check:

○ What are the limitations compared with existing technologies?
○ Could you provide any examples of where you have seen these *problems*?

As we’d like to understand the practical implications of service delivery of FES in the Victorian setting, I would like to drill down to some of the more granular aspects of the current program:

7 Could you please share with me your thoughts around:
  ○ The administration associated with the program
  ○ Referral Process, including liaison with referring clinicians
  ○ MDT review meetings
  ○ Receiving and Reporting of results
  ○ Guidelines and criteria for testing
  ○ Impacts on workload
    - For you and for the healthcare system in general

Check:

○ Could you please give me an example of these experiences?

It would also be helpful to know more about whether the existing program is reaching potential patients, and how accessible the program is.

8 Could you please describe any examples of how well the program has reached the right people? Or possibly ways in which it hasn’t?

As this is an emerging technology, I’d be interested to hear your thoughts about training and support for clinicians. This would include those involved directly with FES such as yourself, as well as those associated with the program such as support staff, referring clinicians, and so on.

9 What feedback do you have about what further clinician training and education could be implemented to improve the FES program?
10 What supports for clinicians would improve the program?

It is possible that the routine clinical use of Fetal Exome Sequencing becomes more widely adopted in Victoria.

11 In addition to what you have already shared, what other changes would you like to see in how Fetal Exome Sequencing is implemented in general?

Finally, thank you for generously responding to my questions.

12 Do you have any questions for me before we finish?

## References

[1] L. Ferretti , R. Mellis , and L. S. Chitty , “Update on the Use of Exome Sequencing in the Diagnosis of Fetal Abnormalities,” European Journal of Medical Genetics 62 (2019): 103663, 10.1016/j.ejmg.2019.05.002. 31085342

[2] M. D. Kilby , “The Role of Next‐Generation Sequencing in the Investigation of Ultrasound‐Identified Fetal Structural Anomalies,” BJOG 128 (2021): 420–429, 10.1111/1471-0528.16533. 32975887 PMC8607475

[3] R. Mellis , K. Oprych , E. Scotchman , M. Hill , and L. S. Chitty , “Diagnostic Yield of Exome Sequencing for Prenatal Diagnosis of Fetal Structural Anomalies: A Systematic Review and Meta‐Analysis,” Prenatal Diagnosis 42 (2022): 662–685, 10.1002/pd.6115. 35170059 PMC9325531

[4] I. B. Van den Veyver , N. Chandler , L. E. Wilkins‐Haug , R. J. Wapner , and L. S. Chitty , “International Society for Prenatal Diagnosis Updated Position Statement on the Use of Genome‐Wide Sequencing for Prenatal Diagnosis,” Prenatal Diagnosis 42 (2022): 796–803, 10.1002/pd.6157. 35583085 PMC11220784

[5] S. Petrovski , V. Aggarwal , J. L. Giordano , et al., “Whole‐Exome Sequencing in the Evaluation of Fetal Structural Anomalies: A Prospective Cohort Study,” Lancet (London, England) 393 (2019): 758–767, 10.1016/s0140-6736(18)32042-7. 30712878

[6] J. Lord , D. J. McMullan , R. Y. Eberhardt , et al., “Prenatal Exome Sequencing Analysis in Fetal Structural Anomalies Detected by Ultrasonography (PAGE): A Cohort Study,” Lancet 393, no. 10173 (2019): 747–757, 10.1016/s0140-6736(18)31940-8. 30712880 PMC6386638

[7] J. Lazier , T. Hartley , J. A. Brock , et al., “Clinical Application of Fetal Genome‐Wide Sequencing During Pregnancy: Position Statement of the Canadian College of Medical Geneticists,” Journal of Medical Genetics 59 (2022): 931–937, 10.1136/jmedgenet-2021-107897. 34544840 PMC9554053

[8] K. G. Monaghan , N. T. Leach , D. Pekarek , P. Prasad , and N. C. Rose , “The Use of Fetal Exome Sequencing in Prenatal Diagnosis: A Points to Consider Document of the American College of Medical Genetics and Genomics (ACMG),” Genetics in Medicine 22, no. 4 (2020): 675–680, 10.1038/s41436-019-0731-7. 31911674

[9] A. Rogers , L. De Jong , W. Waters , et al., “Extending the New Era of Genomic Testing Into Pregnancy Management: A Proposed Model for Australian Prenatal Services,” Australian & New Zealand Journal of Obstetrics & Gynaecology 64 (2024): 467–474, 10.1111/ajo.13814. 38577897 PMC11660018

[10] M. Plantinga , L. Zwienenberg , E. van Dijk , et al., “Parental Experiences of Rapid Exome Sequencing in Cases With Major Ultrasound Anomalies During Pregnancy,” Prenatal Diagnosis 42 (2022): 762–774, 10.1002/pd.6056. 34643287 PMC9298392

[11] E. A. Normand , A. Braxton , S. Nassef , et al., “Clinical Exome Sequencing for Fetuses With Ultrasound Abnormalities and a Suspected Mendelian Disorder,” Genome Medicine 10 (2018): 74, 10.1186/s13073-018-0582-x. 30266093 PMC6162951

[12] F. Mone , H. Abu Subieh , S. Doyle , et al., “Evolving Fetal Phenotypes and Clinical Impact of Progressive Prenatal Exome Sequencing Pathways: Cohort Study,” Ultrasound in Obstetrics & Gynecology: The Official Journal of the International Society of Ultrasound in Obstetrics and Gynecology 59, no. 6 (2022): 723–730, 10.1002/uog.24842. 34940998

[13] C. Deden , K. Neveling , D. Zafeiropopoulou , et al., “Rapid Whole Exome Sequencing in Pregnancies to Identify the Underlying Genetic Cause in Fetuses With Congenital Anomalies Detected by Ultrasound Imaging,” Prenatal Diagnosis 40, no. 8 (2020): 972–983, 10.1002/pd.5717. 32333414 PMC7497059

[14] E. Dempsey , A. Haworth , L. Ive , et al., “A Report on the Impact of Rapid Prenatal Exome Sequencing on the Clinical Management of 52 Ongoing Pregnancies: A Retrospective Review,” BJOG 128 (2021): 1012–1019, 10.1111/1471-0528.16546. 32981126

[15] S. M. Outram , J. E. H. Brown , A. N. Zamora , N. Sahin‐Hodoglugil , and S. L. Ackerman , “Parental Hopes and Understandings of the Value of Prenatal Diagnostic Genomic Sequencing: A Qualitative Analysis,” Frontiers in Genetics 13 (2022): 883225, 10.3389/fgene.2022.883225. 35923691 PMC9339950

[16] K. Wou , T. Weitz , C. McCormack , et al., “Parental Perceptions of Prenatal Whole Exome Sequencing (PPPWES) Study,” Prenatal Diagnosis 38 (2018): 801–811, 10.1002/pd.5332. 30035818

[17] A. N. Talati , K. L. Gilmore , E. E. Hardisty , A. D. Lyerly , C. Rini , and N. L. Vora , “Impact of Prenatal Exome Sequencing for Fetal Genetic Diagnosis on Maternal Psychological Outcomes and Decisional Conflict in a Prospective Cohort,” Genetics in Medicine: Official Journal of the American College of Medical Genetics 23 (2021): 713–719, 10.1038/s41436-020-01025-5. 33214710 PMC8503913

[18] H. McInnes‐Dean , R. Mellis , M. Daniel , et al., “‘Something That Helped the Whole Picture’: Experiences of Parents Offered Rapid Prenatal Exome Sequencing in Routine Clinical Care in the English National Health Service,” Prenatal Diagnosis 44 (2024): 465–479, 10.1002/pd.6537. 38441167

[19] R. Mellis , D. Tapon , N. Shannon , et al., “Implementing a Rapid Fetal Exome Sequencing Service: What Do Parents and Health Professionals Think?,” Prenatal Diagnosis 42 (2022): 783–795, 10.1002/pd.6140. 35383981 PMC9324936

[20] Z. Stark and S. Ellard , “Rapid Genomic Testing for Critically Ill Children: Time to Become Standard of Care?,” European Journal of Human Genetics: EJHG 30 (2022): 142–149, 10.1038/s41431-021-00990-y. 34744166 PMC8821543

[21] L. M. Kelly and M. Cordeiro , “Three Principles of Pragmatism for Research on Organizational Processes,” Methodological Innovations 13, no. 2 (2020): 2059799120937242, 10.1177/2059799120937242.

[22] V. Kaushik and C. A. Walsh , “Pragmatism as a Research Paradigm and Its Implications for Social Work Research,” Social Sciences 8 (2019): 255.

[23] D. F. Vears and L. Gillam , “Inductive Content Analysis: A Guide for Beginning Qualitative Researchers,” Focus on Health Professional Education: A Multi‐Professional Journal 23 (2022): 111–127, 10.11157/fohpe.v23i1.544.

[24] L. Mollison , J. M. O'Daniel , G. E. Henderson , J. S. Berg , and D. Skinner , “Parents' Perceptions of Personal Utility of Exome Sequencing Results,” Genetics in Medicine: Official Journal of the American College of Medical Genetics 22, no. 4 (2020): 752–757, 10.1038/s41436-019-0730-8. 31857707 PMC7192542

[25] I. Bedei , A. Wolter , A. Weber , F. Signore , and R. Axt‐Fliedner , “Chances and Challenges of New Genetic Screening Technologies (NIPT) in Prenatal Medicine From a Clinical Perspective: A Narrative Review,” Genes (Basel) 12 (2021): 12040501, 10.3390/genes12040501. PMC8065512 33805390

[26] M. Peter , R. Mellis , H. McInnes‐Dean , et al., “Delivery of a National Prenatal Exome Sequencing Service in England: A Mixed Methods Study Exploring Healthcare Professionals' Views and Experiences,” Frontiers in Genetics 15 (2024): 1401705, 10.3389/fgene.2024.1401705. 38903755 PMC11188373

[27] S. Narayanan , B. Blumberg , M. L. Clayman , V. Pan , and C. Wicklund , “Exploring the Issues Surrounding Clinical Exome Sequencing in the Prenatal Setting,” Journal of Genetic Counseling 27 (2018): 1228–1237, 10.1007/s10897-018-0245-5. 29525930

[28] N. L. Vora , K. Gilmore , A. Brandt , et al., “An Approach to Integrating Exome Sequencing for Fetal Structural Anomalies Into Clinical Practice,” Genetics in Medicine: Official Journal of the American College of Medical Genetics 22 (2020): 954–961, 10.1038/s41436-020-0750-4. 31974414 PMC7205580

